# Dropwise Evaporative Cooling of Heated Surfaces with Various Wettability Characteristics Obtained by Nanostructure Modifications

**DOI:** 10.1186/s11671-016-1361-5

**Published:** 2016-03-22

**Authors:** Jian-nan Chen, Zhen Zhang, Xiao-long Ouyang, Pei-xue Jiang

**Affiliations:** Key Laboratory for Thermal Science and Power Engineering of Ministry of Education, Beijing Key Laboratory for CO2 Utilization and Reduction Technology, Department of Thermal Engineering, Tsinghua University, Beijing, 100084 China; Institute of Nuclear and New Energy Technology, Tsinghua University, Beijing, 100084 China

**Keywords:** ZnO nanowires, Spray cooling, Droplet, Evaporation, Wettability

## Abstract

A numerical and experimental investigation was conducted to analyze dropwise evaporative cooling of heated surfaces with various wettability characteristics. The surface wettability was tuned by nanostructure modifications. Spray-cooling experiments on these surfaces show that surfaces with better wettability have better heat transfer rate and higher critical heat flux (CHF). Single droplet impingement evaporative cooling of a heated surface was then investigated numerically with various wettability conditions to characterize the effect of contact angle on spray-cooling heat transfer. The volume of fluid (VOF) model with variable-time stepping was used to capture the time-dependent liquid-gas interface motion throughout the computational domain with the kinetic theory model used to predict the evaporation rate at the liquid-gas interface. The numerical results agree with the spray-cooling experiments that dropwise evaporative cooling is much better on surfaces with better wettability because of the better liquid spreading and convection, better liquid-solid contact, and stronger liquid evaporation.

## Background

Gordon Moore proposed that the transistor density on chips will double every 2 years in 1965 [1]. This classical Moore’s Law has been accurate for the last four decades. The tremendous enhancement in chip functionality, e.g., higher transistor density, higher speeds, and more sophisticated functions, have resulted in increasing amounts of heat generated per unit chip surface area. More effective cooling schemes are needed for many industrial applications, such as electronic systems, high-energy lasers, and aerospace satellites. Phase change cooling schemes have attracted the most attention because of the large latent heats of the liquid-vapor phase change. Spray cooling, with its high heat dissipation capability, precise temperature control, low cost, and reliable long-term stability, has played an important role in high heat flux applications as one of the most promising thermal management methods. Heat fluxes in excess of 1000 W/cm^2^ can be removed from surfaces using water spray cooling at low coolant flow rates with low superheats [2].

Spray-cooling heat transfer is influenced by many factors such as the droplet parameters [2, 3], nozzle-to-surface distance [4], inclination angle [5], and working fluid [6]. Surface morphology is another critical factor affecting the spray-cooling heat transfer. Enhanced surfaces, such as millistructured surfaces [7] and micro structured surfaces [8], have been shown to effectively improve the heat transfer. As material science and nanofabrication technologies develop, nanostructured engineering surfaces are becoming more common. Nanostructured surfaces have shown different heat transfer performance for dropwise evaporative cooling. Zhang et al. [9] did spray-cooling experiments with CNT films deposited on surfaces with the heat transfer rate improved by the better wettability. Alvarado and Lin [10] investigated single droplet cooling of nanostructured surfaces and observed lower minimum wall temperatures for similar heat fluxes, better heat transfer curves, and lower temperature gradients on the nanostructured surfaces than those on a bare surface. Thus, nanostructures can improve the heat transfer rate by effectively changing the surface wettability which directly affects the liquid-vapor phase change process. This study experimentally investigates spray-cooling heat transfer on surfaces with various wettability characteristics obtained by nanowire modification.

Spray cooling is affected by many factors with complex heat transfer mechanisms. The droplet parameters are the most important factor. The Sauter mean droplet diameter, the mean droplet velocity, and the droplet density are the three main droplet parameters. The inability to independently control the drop size, drop velocity, and mass flux makes it almost impossible to thoroughly investigate heat transfer mechanism experimentally [7]. Some researchers have studied spray cooling by proposing theoretical and numerical models of single droplet evaporative cooling by independently controlling the droplet parameters numerically. “Before predictions of the heat transfer to a spray can be determined, the fluid dynamics of a single droplet impacting a heated surfaces must be known” [11]. The heat transfer mechanism for how the surface wettability affects the spray cooling can be initially investigated using a numerical model of single droplet impingement cooling of heated surfaces with various wettability characteristics. The flow and evaporation of a single droplet on a surface have been studied extensively with most numerical models based on many assumptions and simplifications of the fluid flow [12, 13], droplet shape [14], and liquid evaporation [15]. The liquid-gas interface tracking method and the liquid-vapor phase change model are key parts of accurate simulations of droplet impingement cooling. In addition to the experimental study of spray cooling on surfaces with different wettabilities, this paper also presents a numerical study of droplet impingement evaporative cooling of surfaces with different wettabilities using the explicit volume of fluid (VOF) model with variable-time stepping to capture the time-dependent liquid-gas interface motion throughout the computational domain and the kinetic theory model to predict the evaporation rate at the liquid-gas interface.

## Methods

## Experimental Investigation

### Spray-Cooling System

**Fig. 1:**
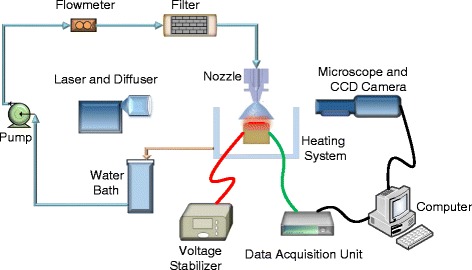
Schematic of spray-cooling system

The experiment system and method presented here were similar to those by Chen et al. [16]. The spray-cooling system shown in Fig. 1 included the spray, heating, and measurement sections. Deionized water was pumped by a Fluid-o-Tech magnetic drive gear pump from a constant temperature water bath through a filter to remove impurities before being sprayed onto the heated surface through a full cone pressure atomizer (Spraying Systems, TG SS 0.3) with a 0.51-mm nozzle orifice. The nozzle was fixed in a bracket with the orifice-to-surface distance adjusted by an accurate micrometer with a positioning accuracy of 0.01 mm. A mechanical pressure gauge was used to measure the nozzle inlet pressure which was assumed to be equal to the spray pressure with an OMEGA 0.125-mm diameter T-type thermocouple imbedded in the flow tube just before the nozzle to measure the deionized water temperature. The experiments used a water spray pressure of 0.3 MPa with an orifice-to-surface distance of 30 mm with subcoolings of approximately −82 to −80 °C.

**Fig. 2:**
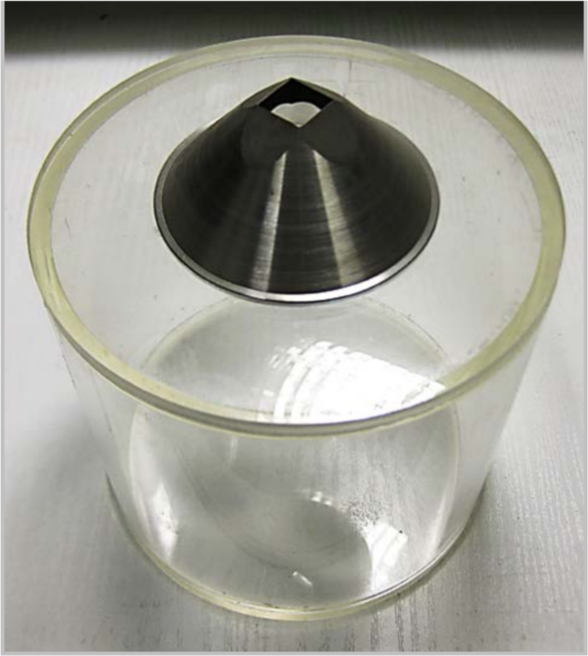
Flow rate measurement container

A flow rate measurement container with a square hole the same size as the heated surface was made to measure the water flow rate impinging the target surface as shown in Fig. 2. The container was first weighed on an electronic scale before the experiments and then fixed under the nozzle instead of the heated surface. The nozzle was adjusted to the same position relative to the hole as for the heated surface. The gear pump was then run for a period of time with the container and the water then weighed to calculate the average flow rate impinging the heated surface. The flow rate was 0.514 kg/m^2^ s for all the spray-cooling experiments.

### Test Heater Fabrication

The heating sections were made of 7.4 mm × 7.4 mm, double-side polished, 490-μm-thick silicon dies. Chromium, platinum, and titanium (thickness proportions of approximately 1:10:1) were applied to the bottom surface of the silicon dies using positive photoresist lift-off in a serpentine pattern with a total thickness of 241 nm. A 150-nm-thick PECVD SiO_2_ film was also added on top of the metals for electrical passivation. Four platinum heaters were arranged in serpentine patterns on the bottom surface of each silicon die to reduce the voltage input for safety considerations with each platinum resistance being 300~400 Ω. The SiO_2_ film and the titanium were removed on the eight pads by wet etching to expose the platinum before the silicon die was mounted on a temperature-resistant PCB circuit board. Eight 40-μm gold wires connected the four platinum heaters with the circuit board by wire bonding. A DC-stabilized voltage source was then used to supply power to the platinum heaters through the eight wires soldered to the circuit board. The currents in each wire were also measured so that the resistances of the platinum heaters could be calculated to determine the heater temperature. Thus, the serpentine platinum heaters acted as the heaters and RTDs. The heat flux was calculated by dividing the input power by the surface area. As the input power increased, surface temperature increased, and at a certain point, test surface temperature increased rapidly and the test sample was burning out. This point was defined as the critical heat flux (CHF) point.

### Test Surface Modification

The surface wettability depends on both the surface morphology and the surface chemical energy, so the surface wettability can be tuned by changing these two parameters. Artificial structures are often fabricated on surfaces to change the surface morphology with nanostructures being especially favored.

ZnO nanowires were synthesized on the surface by hydrothermal methods to modify the surface wettability. Surfaces with different wettabilities were obtained by controlling nanowire size. The basic method and growth mechanisms were described by Xu and Wang [17]. A thin zinc metal film was deposited on top of the silicon die by magnetron sputtering as a seed layer for the ZnO growth. The growth solution was prepared by dissolving zinc nitrate hexahydrate (Zn(NO_3_)_2_ 6H_2_O, 99 %) in deionized water as the source for the ZnO nanowires. Ammonia hydroxide (28 wt% NH_3_ in water, 99.99 %) was added to adjust the pH of the growth solution. Then, the hydrothermal ZnO nanowires were grown by suspending the silicon dies upside down in the growth solution. A variety of parameters such as the Zn^2+^ concentration, the pH, and the growth temperature was tuned to control the properties (mainly the nanowire lengths and diameters of the nanowires) of the final product.

**Fig. 3:**
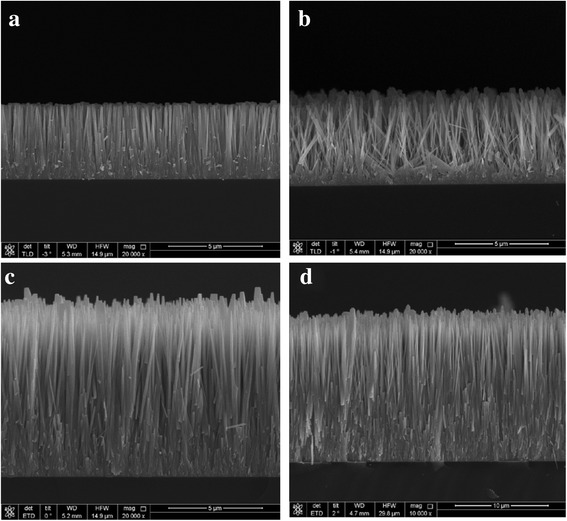
SEM images of the nanowire surfaces: **a** N1 surface, **b** N2 surface, **c** N3 surface, and **d** N4 surface

After the nanowire was grown to change the surface morphology, the surface chemical energy was adjusted by UV illumination. The UV irradiation generated electron-hole pairs in the ZnO nanowire surfaces with some of the electron-hole pairs reacting with lattice oxygen to form surface oxygen vacancies which are kinetically more favorable for hydroxyl adsorption than oxygen adsorption resulting in improved surface hydrophilicity that modifies the surface wettability. Four different wettability surfaces with differently sized nanowires were fabricated for the experiments with the surface morphologies of the nanostructured surfaces imaged by SEM as shown in Fig. 3.

The contact angles of deionized water on the smooth silicon surface and the nanostructured surfaces were measured before the spray-cooling experiments using an Easydrop (Kurss, Germany) with a precision of 0.1°. The results showed that the smooth silicon surface was hydrophilic with a water contact angle of 62.5°. The fabricated nanostructures on the surface enhanced the surface wettability as shown in Table 1.

**Table 1:** Nanowire parameters and contact angles for the different surfaces

Surface	Nanowire length (μm)	Nanowire diameter (nm)	Droplet shape	Contact angle (°)
Smooth	–	–		62.5
N1	4	87		25.0
N2	4	120		9.0
N3	8	180		6.0
N4	16	350		4.6

## Numerical Simulation

### Conservation Equations

The VOF method, developed by Hirt and Nichols [18], is one of the most widely used methods for simulating free surfaces. This is a fixed-mesh method in which the interface between immiscible fluids is modeled as a discontinuity in a characteristic function (such as the volume fraction). In the VOF method, the *q*th-phase volume fraction, *α*_*q*_, is defined as

1 $$ {\alpha}_q=\frac{\mathrm{Volume}\ \mathrm{of}\ \mathrm{fluid}\ q}{\mathrm{Total}\ \mathrm{volume}\ \mathrm{of}\ \mathrm{the}\ \mathrm{control}\ \mathrm{volume}} $$

Then, the following three conditions are possible:

- *α*_*q*_ = 0: The cell is empty (of the *q*th fluid).
- *α*_*q*_ = 1: The cell is full (of the *q*th fluid).
- 0 < *α*_*q*_ < 1: The cell contains the interface between the *q*th fluid and one or more other fluids.

The interface tracking between the phases is accomplished by solving a continuity equation for the volume fraction of one of the phases. For the *q*th phase, this equation has the following form:

2 $$ \frac{1}{\rho_q}\Big[\frac{\partial }{\partial t}\left({\alpha}_q{\rho}_q\right)+\nabla \cdot \left({\alpha}_q{\rho}_q\overrightarrow{v_q}\right)={S}_{\alpha_q} $$

where the volume fractions of all the phases are constrained as ${\displaystyle {\sum}_{q=1}^n{\alpha}_q=1}$. There are only liquid and gas phases in the present study, so *n* = 2. The gas phase is the primary phase, so only the liquid-phase volume fraction, α_l_, is solved. The transport properties in the conservation equations are determined based on the volume fractions of all the phases present in a given computation cell:

3 $$ \rho ={\displaystyle {\sum}_{q=1}^n{\alpha}_q{\rho}_q={\alpha}_l{\rho}_l+\left(1-{\alpha}_l\right){\rho}_g} $$

A single momentum equation is solved throughout the domain with the resulting velocity field shared among the phases.

4 $$ \frac{\partial }{\partial t}\left(\rho \overrightarrow{v}\right)+\nabla \cdot \left(\rho \overrightarrow{v}\overrightarrow{v}\right)=-\nabla p+\nabla \cdot \left[\mu \left(\nabla \overrightarrow{v}+\nabla {\overrightarrow{v}}^T\right)\right]+\rho \overrightarrow{g}+\overrightarrow{F}+{S}_m $$

**Fig. 4:**
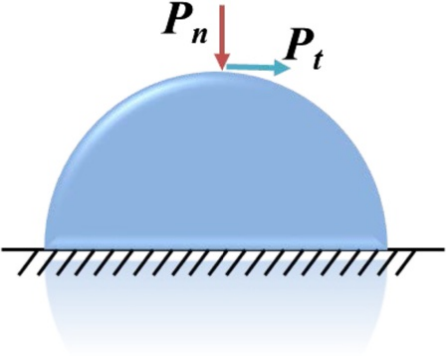
Schematic of the capillary pressure and the surface tension gradient pressure at the interface

The source term, *S*_*m*_, in the equation is because of the mass transfer at the liquid-gas interface due to evaporation. $\overrightarrow{F}$ is the surface tension body force (which is nonzero only at the free surface). $\overrightarrow{F}$ is composed of a normal force induced by the pressure jump due to surface tension and a tangential force due to surface tension gradient along the free surface as shown in Fig. 4. The capillary pressure is given by the Young-Laplace equation as

5 $$ {P}_n=\sigma \cdot \kappa $$

The pressure caused by the surface tension gradient along the free surface is given as

6 $$ {P}_t={\nabla}_t\sigma $$

Referring to the continuous surface force (CSF) model, $\overrightarrow{F}$ is calculated as

7 $$ \overrightarrow{F}=\frac{\left|\nabla {\alpha}_l\right|\rho }{\frac{1}{2}\left({\rho}_l+{\rho}_g\right)}\left(\sigma \kappa \widehat{n}+{\nabla}_t\sigma \right) $$

The energy equation, which is also shared among the phases, is

8 $$ \frac{\partial }{\partial t}\left(\rho E\right)+\nabla \cdot \left(\overrightarrow{v}\left(\rho E+p\right)\right)=\nabla \cdot \left(\lambda \nabla T\right)+{S}_E $$

where the energy, *E*, is the mass-averaged value as shown below:

9 $$ E=\frac{{\displaystyle {\sum}_{q=1}^n}{\alpha}_q{\rho}_q{E}_q}{{\displaystyle {\sum}_{q=1}^n}{\alpha}_q{\rho}_q} $$

Liquid evaporation often occurs at the interface as the water droplets evaporate into the air. The gas phase is then a binary mixture of vapor and air. The vapor diffusion and convection in the air must be described to accurately model the evaporation process. Thus, the species transport equation was solved in the gas phase with the VOF model

10 $$ \frac{\partial \left({\rho}_q{\alpha}_q{y}_q^i\right)}{\partial t}+\nabla \cdot \left({\rho}_q{\alpha}_q\overrightarrow{v}{y}_q^i\right)=\nabla \cdot \left({\rho}_q{\alpha}_q{D}_{AB}\nabla {y}_q^i\right)+{S}_i $$

where ${y}_q^i$ denotes the mass fraction of the *i*th species in the *q*th phase.

## Interface Conditions

The mass transfer at a liquid-gas interface is essentially due to molecular movement. Above absolute zero temperature, every molecule has a certain kinetic energy which is directly related to the temperature. The kinetic energies of the molecules in a liquid are not the same for all molecules. Molecules in the liquid tend to remain together because they are bound to their neighbors by intermolecular forces. At a liquid and air interface, the liquid molecules have less neighbors than those inside the liquid. Therefore, the bond to their neighbors is not as strong as that inside the liquid. Molecules with relatively high kinetic energies can then escape from the liquid into the air. Molecules in the vapor phase move at relatively high velocities and sometimes collide with each other. Some of the vapor molecules may also return into the liquid when they collide with the interface while others are reflected back into the air. Evaporation occurs when the number of the molecules escaping from the liquid is larger than the number of molecules entering the liquid.

The mass flux of molecules impacting the liquid surface is *J*_*i*_; however, only a proportion of these molecules, *J*_*c*_, actually condense into the liquid. The remainder of the molecules, *J*_*r*_, rebound from the surface without entering the liquid. The mass flux of molecules evaporating from the surface is *J*_*e*_, so the net mass flux of liquid evaporation is

11 $$ {J}_t={J}_e-{J}_c={J}_e-{\sigma}_c{J}_i $$

According to the kinetic theory proposed by Schrage [19], the flux of molecules impacting the liquid surface is given by

12 $$ {J}_i=\left(1-a\sqrt{\pi}\right)\sqrt{\frac{M}{2\pi R}}\frac{P_v}{\sqrt{T_g}} $$

where $a={u}_g\sqrt{\frac{M}{2R{T}_g}}$ and *u*_*g*_ is the evaporating gas velocity.

Assuming equilibrium at the interface, the mass flux of molecules evaporating from the surface is given by

13 $$ {J}_e={\sigma}_c\sqrt{\frac{M}{2\pi R}}\frac{P_{\mathrm{sat}}\left({T}_l\right)}{\sqrt{T_l}} $$

Then, the total evaporating mass flux at a liquid-gas interface is

14 $$ {J}_t=\frac{2{\sigma}_c}{2-{\sigma}_c}\sqrt{\frac{M}{2\pi R}}\left(\frac{P_{\mathrm{sat}}\left({T}_l\right)}{\sqrt{T_l}}-\frac{P_v}{\sqrt{T_g}}\right) $$

The VOF model assumes that *T*_*l*_ = *T*_*g*_ = *T* (temperature of the cell). *P*_*v*_ is the vapor partial pressure in the cell and *P*_sat_(*T*_*l*_) is the saturation pressure at *T. J*_*t*_ denotes the evaporation rate per unit area with the evaporation rate per unit cell then calculated as

15 $$ {m}^{{\prime\prime\prime} }=\left|\nabla {\alpha}_l\right|\cdot {J}_t=\left|\nabla {\alpha}_l\right|\frac{2{\sigma}_c}{2-{\sigma}_c}\sqrt{\frac{M}{2\pi RT}}\left({P}_{\mathrm{sat}}(T)-{P}_v\right) $$

The droplet evaporation results in discontinuities at the liquid-gas interface which are implemented as the source terms in the conservation equations listed in Table 2. The source terms are nonzero only at the liquid-gas interface and zero in the rest of the flow domain.

**Table 2:** Source terms appearing in conservation equations

Equations	Source terms
VOF (liquid phase)	*S*_*αl*_ = −*m*′′′
VOF (gas phase)	*S*_*αg*_ = *m*′′′
Momentum	${S}_m=\left(1-2{\alpha}_l\right){m}^{\prime \prime \prime}\overrightarrow{v}$
Energy	*S*_*e*_ = −*m*′′′*h*_*fg*_
Species (vapor)	*S*_*i*_ = *m*′′′

## Solution Method

The commercial CFD software Ansys Fluent 13 was used to perform the numerical simulations. Ansys Fluent uses the control volume method to discretize the governing equations on an unstructured grid. The second-order upwind scheme was used to discretize the transport equations. The pressure values at the cell faces were obtained using the PRESTO! discretization scheme. The SIMPLEC algorithm was used for pressure-velocity coupling.

The properties of the pure species (liquid, air, and vapor) were assumed to be functions of temperature and, thus, were updated at every time step. The binary diffusion coefficient was defined as below [20]:

16 $$ D=\frac{10^{-3}{T}^{1.75}{\left(\frac{1}{M_1}+\frac{1}{M_2}\right)}^{\frac{1}{2}}}{P{\left[{\left({\displaystyle {\sum}_i}{V}_{i1}\right)}^{\frac{1}{3}}+{\left({\displaystyle {\sum}_i}{V}_{i2}\right)}^{\frac{1}{3}}\right]}^2} $$

## Numerical method validation

**Fig. 5:**
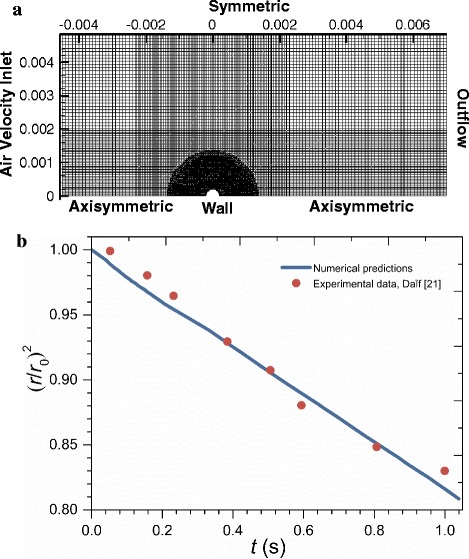
**a** Schematic of the flow domain for the validation case; **b** comparison of the predicted and measured droplet sizes

The numerical model was validated against experimental data (DaÏf et al., [21]). A suspended liquid droplet of n-heptane with an initial temperature of 302 K was left to vaporize in air at 356 K temperature at atmospheric pressure. The initial droplet radius was 0.526 mm, while the free-stream gas-phase axial velocity and temperature were 3.2 m/s and 356 K, respectively. Figure 5a shows a schematic of the flow domain with the computational grid and boundary conditions for the validation simulation. The mesh had 73,269 cells with a maximum element size of 80 μm × 80 μm. Three levels of local refinement were then used with the element in the inner region containing the droplet being 8 μm × 8 μm.

The numerical results are compared against experimental data in Fig. 5b. The numerical model accurately predicts the evaporation process, so this model can be used to simulate droplet flow dynamics, heat transfer, and evaporation.

## Results and discussion

## Experimental Results

**Fig. 6:**
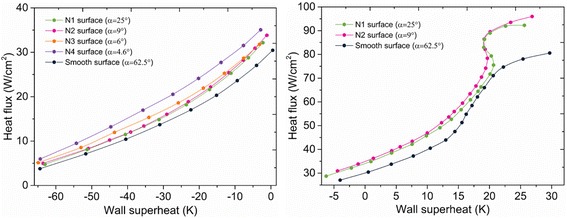
Heat transfer rates on surfaces with different wettabilities

The measured heat transfer rates in spray-cooling experiments on the different wettability surfaces are shown in Fig. 6. The heat transfer curves are plotted on two figures with the spray-cooling heat transfer subdivided into two regimes based on the wall superheat. At low heat fluxes and wall temperatures far below saturation, the heat transfer mainly depends on the forced convection induced by the droplet impacts with little evaporation. At higher heat fluxes and wall superheats, liquid evaporation becomes stronger so the heat transfer is enhanced. The surfaces with better wettabilities have better heat transfer rates for all conditions and higher CHF, most likely due to the spray droplets spreading faster and thinner liquid films on the hydrophilic surfaces which result in better heat transfer and promote evaporation. Kim et al. [22] reported that surface nanostructures may enlarge the liquid contact area to improve the heat transfer while capillary wicking on the nanostructured surface may also enhance liquid transport on the surface that improves CHF in pool boiling. These effects may also exist in the present spray-cooling tests. However, this paper focuses on the wettability effect. The surface nanostructures enlarged the contact area so that the Wenzel state was reached to make the surface hydrophilic. Capillary wicking also leads to smaller contact angles on the surfaces. Thus, the nanostructure effect is strongly related to the wettability changes which can be modeled numerically. Nanostructures cannot be modeled in macro scale simulations, so the surfaces were modeled as smooth walls with different contact angles to account for the wettability effects in the following numerical analyses.

## Simulation Results and Discussions

**Fig. 7:**
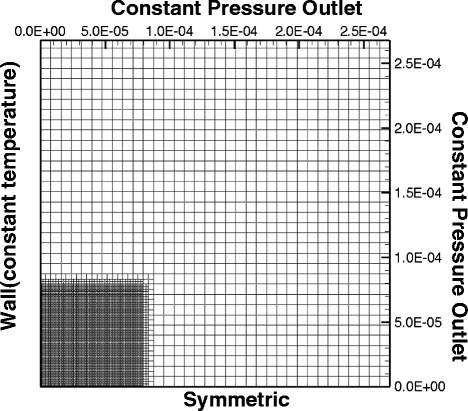
Schematic of the flow domain, mesh, and boundary conditions for the simulations

Single water droplet impingement cooling of different wettability surfaces was numerically simulated to provide more insight into the spray-cooling experiments. The droplet analysis by the shadowgraph technique showed that the mean droplet diameter of the spray in the experiments was 53.4 μm and the droplet velocity was at least 5 m/s. Thus, the simulation used a 60-μm-diameter micro-sized droplet with an initial velocity of 5 m/s. The impingement cooling was simulated on a 2D axisymmetric grid as shown in Fig. 7. The base element in the outer part of the mesh was 8 μm × 8 μm. Grid independence tests gave a refinement level of 3 for the simulations considering the simulation accuracy and computation cost. Six wall surfaces with different wettabilities were used in the simulations with contact angles with water of 5°, 15°, 30°, 60°, 85°, and 155°. The water droplet was initially 2 μm above the wall surface. The wall temperature was 373.15 K, and the initial temperatures of the water droplet and the surrounding air were 293.15 K.

**Fig. 8:**
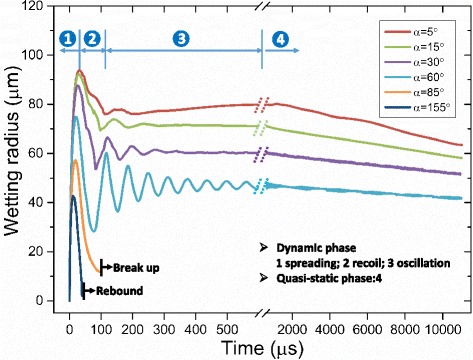
Effect of surface wettability on the spread of a droplet during impingement cooling

Figure 8 shows the spreading radius of the water droplet on surfaces with different wettabilities during impingement cooling. As in previous droplet impact dynamics studies [23, 24], the impingement cooling process was subdivided into a dynamic phase and a quasi-static phase. The horizontal axis is split and scaled to clearly show the very short dynamic-phase characteristics along with the longer quasi-static-phase characteristics in the same figure. When the droplet just touches the surface, the droplet rapidly spreads radially. After the fluid has reached its maximum radial extent, there may be rapid recoil followed by a relatively long period of damped oscillations. The droplet then comes to rest after its excess energy is dissipated and the quasi-static evaporation phase is reached. In the quasi-static phase, the droplet lies quietly on the surface as its volume slowly decreases due to evaporation with little change in the droplet shape. Therefore, the wetting radius slowly decreases in the quasi-static phase.

Weber number and Ohnesorge number are often used to analyze droplet impact dynamics. Droplet impact dynamics is classified into four different regimes according to We and Oh [25]. In regime I, where We > 1 and Oh < 1, kinetic energy dominant motion prevails; in regime II, where We < 1 and Oh < 1, capillary force drives the motion; in regime III, where We < 1 and Oh > 1, capillary effect is dominant and the viscosity is also important; and in regime IV, where We > 1 and Oh > 1, kinetic energy dominates and the viscous force is important as well. In the present simulation, We = 23 and Oh = 0.0086, droplet impact dynamics is in regime I. As kinetic energy dominates in regime I, droplets may spread, recoil, oscillate, and even rebound depending on the surface characteristics.

**Fig. 9:**
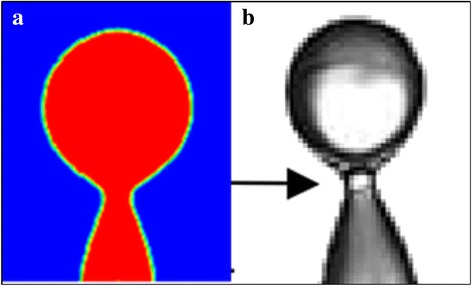
Droplet breakup phenomenon: **a** numerical results; **b** experiment observation [23]

**Fig. 10:**
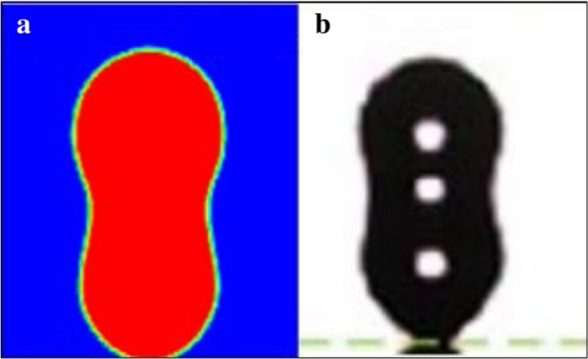
Droplet rebound phenomenon: **a** numerical results; **b** experiment observation [24]

As can be seen from Fig. 8, surfaces with better wettability have larger maximum spreading radii, less recoil, and weaker oscillations which result in a larger wetting area throughout the impingement cooling process, especially during the long quasi-static evaporation phase. Table 3 shows the droplet shapes on different surfaces at different times. When all the droplets reach their maximum spreading radii at about 40 μs, the droplet on the 5° contact angle surface has the largest spreading radius of 93 μm and the thinnest liquid film while the maximum spreading radius for the droplet on the 60° contact angle surface is 74 μm. All the droplets recoil and reach their minimum contact radii during the dynamic phase at about 85 μs. The minimum contact radius during the dynamic phase for the droplet on the 5° contact angle surface is 75 μm which means that the outer radius recoils 18 μm while the minimum contact radius during the dynamic phase for the droplet on the 60° contact angle surface is 28 μm with the radius recoiling by 46 μm. The simulation results agree with experiment results of Šikalo et al. [23] that a droplet takes longer to reach a final state on a surface with worse wettability. The poor wettability surface which corresponds to a contact angle larger than 85° in the present simulations causes the impacting droplet to break up while partially rebounding from the surface at 95 μs as shown in Fig. 9a or to completely rebound from the surface at 50 μs as shown in Fig. 10a. Experiment observations of Šikalo et al. [23] and Kim et al. [24] also validate the simulated phenomena. The droplet Weber numbers in Šikalo’s experiment and Kim’s experiment are 90 and 4.6, respectively, and the droplet Ohnesorge numbers in Šikalo’s experiment and Kim’s experiment are 0.0019 and 0.00028, respectively. So the droplets in their experiments and the simulated droplets are in the same regime which makes them show similar impact dynamics. In Šikalo’s experiment, the droplet broke up (as shown in Fig. 9b) when impacting a 95° contact angle wax surface. In Kim’s experiment, the droplet completely rebounded (as shown in Fig. 10b) when impacting a 160° contact angle nanostructured surface. As illustrated in Figs. 9 and 10, the simulated phenomena agree well with the experiment observations of Šikalo et al. and Kim et al. that impacting droplets tend to rebound (partially or completely) from surfaces with poor wettabilities but more easily deposit on surfaces with better wettabilities.

**Table 3:** Temporal evolution of the droplet shapes on surfaces with different wettabilities during impingement cooling

	*α* = 5°	*α* = 30°	*α* = 60°
40 μs			
85 μs			
1500 μs			
10,000 μs

**Fig. 11:**
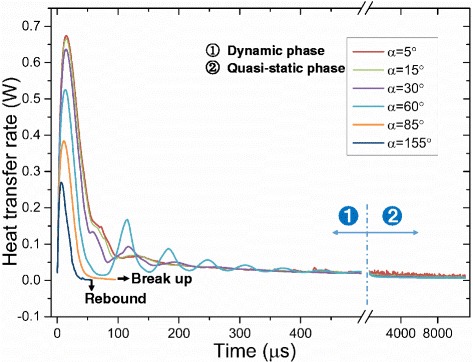
Heat transfer rate on surfaces with different wettabilities

**Fig. 12:**
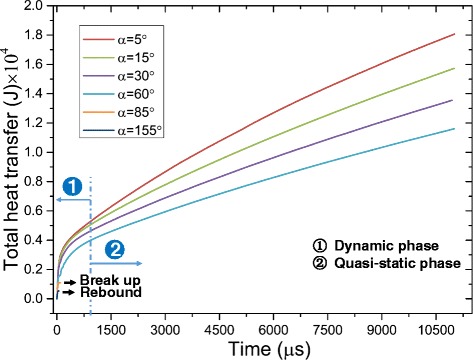
Total heat transfer on surfaces with different wettabilities

**Fig. 13:**
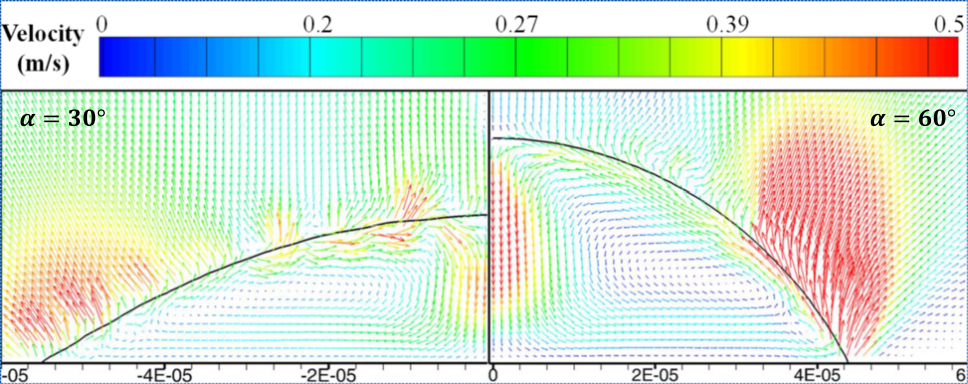
Velocity vector plot for droplets on the 30° contact angle surface and the 60° contact angle surface at *t* = 5505 μs

Figures 11 and 12 show the heat transfer for droplet impingement cooling on surfaces with different wettabilities. The total heat transfer is the integral of the heat transfer rate over time. In the first phase, the dynamic phase, the heat transfer is dominated by forced convection and less than 1 % of the total volume evaporates during the dynamic phase. There is strong liquid flow and large temperature gradients during the droplet spreading, so the heat transfer rate is very large. However, after spreading, the liquid kinetic energy dissipates and the convection slows, so the heat transfer rate decreases rapidly. When the impacting droplet comes to rest on the surface, the quasi-static phase begins and liquid evaporation dominates the heat transfer. However, even after the droplet comes to rest on the surface, there is still some circulation in the droplet mainly due to the liquid evaporation and the Marangoni effect. Figure 13 shows the velocity vectors inside and outside the droplet at 5505 μs which is in the quasi-static phase. The black solid line depicts the droplet shape obtained from the liquid volume fraction of 0.5. The liquid evaporation is stronger at the edge of the droplet because the temperature at the edge is higher than that at the top of the droplet. The temperature difference along the droplet surface produces a surface tension gradient and this thermocapillary force induces the Marangoni convection. Marangoni convection inside the droplet improves the heat transfer and the heat transfer uniformity in the quasi-static phase.

The heat transfer rate is relatively small in the quasi-static evaporation phase compared to that in the dynamic forced convection phase as can also be seen in Fig. 12 when the slopes of the curves during the dynamic phase are larger than those in the quasi-static phase. The total heat transfer in the dynamic phase, however, is not large because the spreading lasts only tens of microseconds with more heat removed during the relatively long evaporation. This result can be used to explain the Estes and Mudawar spray-cooling experiment [3] where the evaporation efficiency was higher with light sprays than with dense sprays, since the droplet evaporation needs a relatively long time and the droplets evaporate more in light sprays by avoiding the frequent impacts of other droplets.

Droplet impingement cooling on surfaces with better wettabilities has much better heat transfer rates as seen in Figs. 11 and 12. During the dynamic phase, the with better wettability has faster liquid spreading, stronger convection, and larger heat transfer areas that all increase the heat transfer.

**Fig. 14:**
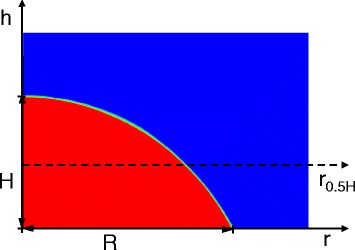
Schematic of parameters for the Marangoni effect analysis

**Fig. 15:**
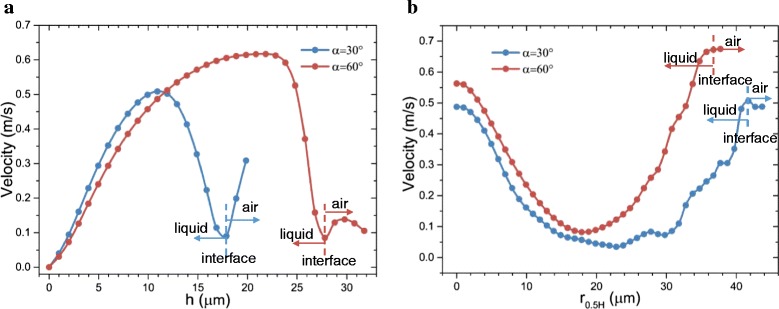
Velocity profile comparison inside the droplet at *t* = 5505 μs: **a** velocity profile at *h* = *r*_0.5*H*_; **b** velocity profile at *r* = 0

As the impact kinetic energy dissipates, the impinging droplet becomes almost stationary with the Marangoni effect driving circulation inside the droplet. Considering the typical geometry in Fig. 14, droplets on surfaces with different wettabilities have different shapes and different Marangoni convection flows. The velocity vector plots in Fig. 13 show that the Marangoni convection is stronger inside the droplet on the 60° contact angle surface than that on the 30° contact angle surface. Figure 15 compares the velocity profiles for droplets on two surfaces with different wettabilities along two different lines. The circulation velocities are larger for the droplet on the 60° contact angle surface than that on the 30° contact angle surface.

A scale analysis showed how the surface wettability affects the Marangoni convection. The modified Marangoni velocity scale, *U*_Ma_, was obtained based on a scale analysis by balancing the tangential stress at the interface of the droplet with the thermocapillary stress:

17 $$ \mu \frac{U_{\mathrm{Ma}\ }}{H}=\frac{\partial \sigma }{\partial T}\cdot \frac{\varDelta T}{R} $$

18 $$ {U}_{\mathrm{Ma}}=\frac{\sigma_T\varDelta T}{\mu}\cdot \frac{H}{R} $$

where *H* is the droplet height, *R* is the wetting radius, ${\sigma}_T=\frac{\partial \sigma }{\partial T}$, and *ΔT* represent a characteristic temperature difference.

Assuming that the droplet shape is a spherical cap:

19 $$ \frac{H}{R}=\frac{1- \cos \alpha }{ \sin \alpha } $$

where *α* is the liquid-solid contact angle. Substituting Eq. (19) in Eq. (18) gives

20 $$ {U}_{\mathrm{Ma}}=\frac{\sigma_T\varDelta T}{\mu}\cdot \frac{1- \cos \alpha }{ \sin \alpha } $$

**Fig. 16:**
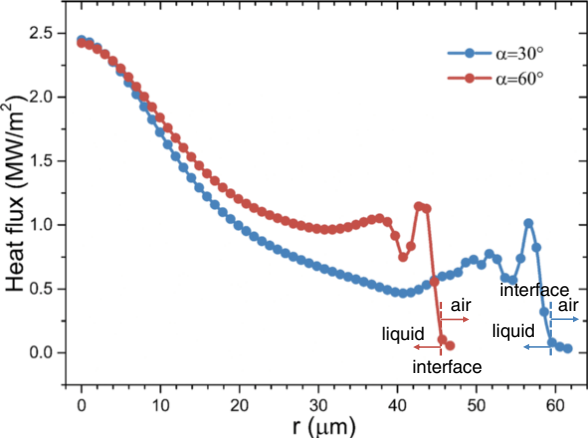
Substrate heat flux distribution comparison at *t* = 5505 μs

A modified Marangoni number is then defined based on this reference velocity:

21 $$ \mathrm{Ma}=\frac{U_{\mathrm{Ma}}{R}_0}{\kappa }=\frac{\sigma_T\varDelta T{R}_0}{\mu \kappa}\cdot \frac{1- \cos \alpha }{ \sin \alpha } $$

where *R*_0_ is the initial droplet radius and *κ* is the liquid thermal diffusivity. From Eqs. (20) and (21), as the surface wettability decreases and *α* increases, *U*_Ma_ and Ma both increase. This agrees with the simulation results in Fig. 13 that Marangoni convection is stronger for droplets on a surface with larger contact angles. When the Marangoni convection is stronger, the local heat transfer is also better for surfaces with larger contact angles during the quasi-static phase as seen in Fig. 16.

**Fig. 17:**
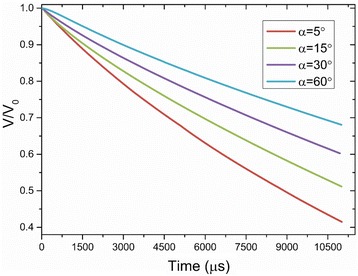
Temporal evolution of the droplet volume for impingement cooling on surfaces with different wettabilities

However, as mentioned before, better surface wettability results in larger wetting radii during the quasi-static phase. This means that the liquid films formed on surfaces with better wettabilities have larger liquid-solid contact areas and larger liquid-vapor interfacial areas which both increase the liquid evaporation. Thus, the cooling during the quasi-static phase on surfaces with better wettabilities is still better as can be seen from Fig. 12 where the slopes of the curve for surfaces with better wettabilities are larger during the quasi-static phase. Figure 17 shows that droplets evaporate much more quickly on surfaces with better wettabilities. ${t}_{\frac{1}{2}}$ for a droplet on the surface with a 5° contact angle is about 9 ms while ${t}_{\frac{1}{2}}$ for a droplet on a 60° contact angle surface is about 25 ms. This is also the reason why the wetting radius decreases more quickly for droplets on surfaces with better wettabilities during the quasi-static phase as shown in Fig. 8. Table 3 shows the droplet shapes on different surfaces at different times as the droplets spread, recoil, come to rest on the surface, and evaporate for relatively long times to further clarify the analysis.

The numerical results agree well with spray-cooling experiments which show that dropwise evaporative cooling on surfaces with better wettabilities is much better because of the better liquid spreading and convection, better liquid-solid contact, and stronger liquid evaporation. The droplet impingement cooling simulations reveal the mechanisms for spray cooling of surfaces with various wettabilities based on simulations of a single droplet.

## Conclusions

Nanostructures effectively change the surface wettability which directly affects the liquid-vapor phase change process. The spray-cooling heat transfer of surfaces with different wettability characteristics obtained by nanostructure surface modifications is experimentally studied in this work. The heat transfer mechanisms were then studied using simulations of single droplet impingement cooling on heated surfaces with various wettabilities. Some major observations from this study are:

1. Nanostructures significantly affect the surface wettability. The spray-cooling experiments show that surfaces with better wettability have higher heat transfer rates during the whole process and higher CHF. The experiments indicate that both the liquid forced convection and the liquid evaporation are enhanced for surfaces with better wettability.
2. The numerical simulations agree well with the spray-cooling experiments which show that the dropwise evaporative cooling on surfaces with better wettability are much better because of the better liquid spreading and convection, better liquid-solid contact, and stronger liquid evaporation.
3. The numerical simulations give more detailed evidence to explain the experiment results. The droplet impingement cooling process is subdivided into a dynamic phase and a quasi-static phase. Surfaces with better wettabilities have faster spreading and larger maximum spreading radii which result in stronger liquid convection during the dynamic phase. Droplets on surfaces with worse wettabilities recoil more after their maximum spreading radii and even rebound from the surface. Surfaces with better wettabilities have large liquid wetting areas during the quasi-static phase, so the liquid evaporation is increased because of larger liquid-vapor interfaces and thinner liquid films.
4. Marangoni convection in the droplets on surfaces with different wettabilities was also studied in the numerical simulations. A modified Marangoni number was presented to explain the simulation results showing that Marangoni convection is stronger inside droplets on a surface with smaller contact angles.

## Nomenclature

*CHF* critical heat flux

*D* binary diffusion coefficient

*D*_0_ droplet diameter

*E* energy

$\overrightarrow{F}$ surface tension body force

*J*_*i*_ mass flux of molecules impacting the liquid surface

*J*_*c*_ mass flux of molecules condensing onto the liquid

*J*_*r*_ mass flux of molecules rebounding from the surface

*J*_*e*_ mass flux of molecules evaporating from the surface

*J*_*t*_ net liquid evaporation mass flux

*m*′′′ evaporation rate of unit cell

*Ma* Marangoni number

*Oh* Ohnesorge number = $\mu /\sqrt{D_0\sigma \rho }$

*P*_*v*_ vapor partial pressure

*P* pressure

${S}_{\alpha_q}$ VOF equation source term

*S*_*m*_ momentum equation source term

*S*_*E*_ energy equation source term

*S*_*i*_ species equation source term

*T* cell temperature

${t}_{\frac{1}{2}}$ droplet half lifetime

*U*_Ma_ Marangoni velocity scale

*U*_0_ droplet impact velocity

*We* Weber number = $\rho {D}_0{U}_0^2/\sigma$

*y* mass fraction

*Greek symbols*

*α* volume fraction

*σ* surface tension

*μ* viscosity

*ρ* density

*Subscripts*

*n* normal vector

*q* the *q*th phase

*t* tangential vector
